# Dearth and Delayed Maturation of Testicular Germ Cells in *Fanconi Anemia E* Mutant Male Mice

**DOI:** 10.1371/journal.pone.0159800

**Published:** 2016-08-03

**Authors:** Chun Fu, Khurshida Begum, Philip W. Jordan, Yan He, Paul A. Overbeek

**Affiliations:** 1 Department of Obstetrics and Gynecology, Second Xiangya Hospital, Central South University, Changsha, 410011, China; 2 Department of Molecular and Cellular Biology, Baylor College of Medicine, Houston, TX, 77030, United States of America; 3 Department of Biochemistry and Molecular Biology, Johns Hopkins Bloomberg School of Public Health, Baltimore, MD, 21205, United States of America; China Agricultural University, CHINA

## Abstract

After using a self-inactivating lentivirus for non-targeted insertional mutagenesis in mice, we identified a transgenic family with a recessive mutation that resulted in reduced fertility in homozygous transgenic mice. The lentiviral integration site was amplified by inverse PCR. Sequencing revealed that integration had occurred in intron 8 of the mouse *Fance* gene, which encodes the Fanconi anemia E (Fance) protein. Fanconi anemia (FA) proteins play pivotal roles in cellular responses to DNA damage and Fance acts as a molecular bridge between the FA core complex and Fancd2. To investigate the reduced fertility in the mutant males, we analyzed postnatal development of testicular germ cells. At one week after birth, most tubules in the mutant testes contained few or no germ cells. Over the next 2–3 weeks, germ cells accumulated in a limited number of tubules, so that some tubules contained germ cells around the full periphery of the tubule. Once sufficient numbers of germ cells had accumulated, they began to undergo the later stages of spermatogenesis. Immunoassays revealed that the Fancd2 protein accumulated around the periphery of the nucleus in normal developing spermatocytes, but we did not detect a similar localization of Fancd2 in the *Fance* mutant testes. Our assays indicate that although *Fance* mutant males are germ cell deficient at birth, the extant germ cells can proliferate and, if they reach a threshold density, can differentiate into mature sperm. Analogous to previous studies of FA genes in mice, our results show that the Fance protein plays an important, but not absolutely essential, role in the initial developmental expansion of the male germ line.

## Introduction

Fanconi anemia (FA) patients exhibit a syndrome characterized by bone marrow failure, congenital malformations and cancer predisposition. The phenotypic manifestations of FA are thought to be related to deficiencies in DNA damage repair [1–3]. Interestingly, mice with mutations in FA genes have shown phenotypes that are somewhat different from the human patients. For example, gonadal abnormalities and reduced fertility have been observed in all FA mouse models described thus far [4–12]. In addition to germ cell deficits, adult FA mutant male mice have been found to exhibit Leydig cell hyperplasia and vacuolization of Sertoli cells in the testes [9, 13].

The Fance protein is thought to act as a molecular bridge linking the FA core complex to Fancd2[14]. The Fancm protein recognizes stalled replication forks, then activates the FA core complex (comprised of at least 8 FA proteins, including Fanca, -b, -c, -e, -f, -g, -l, and -m) to monoubiquitinate Fancd2-Fanci, and to trigger the downstream machinery for DNA repair [3, 15, 16]. To date, research has not been done on a *Fance* mutant animal model [17, 18]. We used a self-inactivating lentivirus containing a tyrosinase minigene to infect preimplantation FVB mouse embryos. Stable, single-copy, integration of the lentivirus into the genome results in expression of functional tyrosinase protein and rescue of albinism, so transgenic mice can be identified by simple visual inspection. After inbreeding a cohort of transgenic families, we identified a family with reduced fertility in the offspring that were homozygous for the lentiviral transgene. We amplified the lentiviral integration site by inverse PCR. Sequencing of the PCR products revealed that the lentivirus had integrated into intron 8 of *Fance*. We describe herein our characterization of hypogonadism and germ cell deficiencies in *Fance* mutant male mice.

## Materials and Methods

### Animals, genotype analysis and phenotypic observations

Mice were housed and bred under specific-pathogen-free conditions in the mouse breeding facility at the Baylor College of Medicine. Mouse experiments were performed with the approval of the Institutional Animal Care and Use Committee in accordance with institutional guidelines. All mice were maintained on the inbred FVB/N background. FVB/N mice are normally albino due to a mutation in their tyrosinase gene, and this strain has a number of traits that are advantageous for transgenic research, including excellent fertility [19, 20]. Mice were sacrificed by CO_2_ inhalation when necessary.

A self-inactivating lentivirus carrying a tyrosinase miningene was packaged and used to generate transgenic mice by injection under the zona pellucida of FVB/N two-cell stage mouse embryos. Potential founders were identified by inspection for pigmentation. Expression of the tyrosinase minigene results in melanin production and gene therapy for albinism. Founder mice were bred to albino FVB/N mice and F1 offspring with distinct coat colors (i.e., with different sites of integration in the genome) were used to establish transgenic lines. New lines were inbred to generate homozygous transgenic mice, and the homozygotes were assessed for altered phenotypes. The F0 mouse for line OVE2364 was a male with dark grey, mottled, pigmentation. His pigmented F1 offspring showed coat colors ranging from light tan to dark grey. The F1 mice were bred to albino FBV/N partners to generate lines OVE2364A-F. The 2364E F1 mouse generated offspring with 3 different coat colors. Based upon coat color segregation and inverse PCR assays, the OVE2364E-2 mice were found to have two single copy lentiviral insertion sites. One integration site was located in intron 1 of the *Vkcor1l1* gene on chromosome 5 (data not shown). The other integration site mapped to intron 8 of the *Fance* gene on chromosome 17 [21]. Using PCR primers specific for these integration sites, line OVE2364E-2a2 was established and shown to carry only the *Fance* insertion site. This line has been maintained by brother-sister matings for more than 4 years. In each generation, the coat color phenotype has matched the PCR-based genotyping results, consistent with the presence of a single copy insertion at a single site in the genome.*Fance* mutant males were genotyped using primers LF (left flank) 5’-tggcatctccacttctctatca and RF (right flank) 5’-agagcagcctggactacttgag. Mice with a wild type copy of *Fance* intron 8 produced an amplification band of 620 bp, while the homozygous mutants showed no amplification band (due to the presence of the intervening lentiviral sequences). Genomic DNA was extracted from tail snips of 1- to 3-week-old mice.

Body weights were recorded weekly and testes were harvested from mice at 1 week to 8 weeks of age. We found no apparent phenotypic differences between wild type mice (WT, *Fance*^+/+^) and heterozygous mice(*Fance*^+/-^), other than their coat color. For the comparative fertility assays, the mice were divided into 2 groups: one containing WT or *Fance*^+/-^ males and the other containing homozygous mutant males (*Fance*^-/-^). Each group consisted of 8 mice. Each male mouse was paired with one or two female mice (*Fance*^+/+^ or *Fance*^+/-^) in one cage. The male mice were 8-weeks-old and the female mice were 6-weeks-old. Cages were monitored daily, and pregnancies and births were recorded for a 6-month period.

### RNA expression analysis

Total RNA was extracted by RNeasy Mini Kit (Cat# 74104, Qiagen) and cDNA was synthesized using the SuperScriptTMIII first-strand synthesis system (Cat# 18080–051, Invitrogen^™^). The *Fance* cDNA from exon 6 to exon 10 was amplified using a sense primer (5’-cctcgtctccttctgtgtaaagt) and an antisense primer (5’-tgtgttctgagacaagcagtcag). The hypoxanthine phosphoribosyltransferase (Hprt) transcript was amplified using sense primer 5’-atgacctagatttgttttgtatacc and antisense primer 5’-gtagctcttcagtctgataaaatctac, as a control.

Real-time PCR amplification was performed with the PerfeCTa^®^ SYBR^®^ Green SuperMix (Cat# 95055–100, Quanta BioSciences) and the StepOnePlus real-time PCR system (Applied Biosystems). The primers for real time RT-PCR were the same as the RT-PCR primers. Relative transcript levels were obtained by subtracting the threshold cycle (Ct) value of Hprt from the corresponding Ct value of *Fance*. Differences in mRNA levels were calculated based on △△Ct; the relative copy number then is 2^-△△Ct^.

In situ hybridizations were done in the In Situ Hybridization Core Lab of the Baylor College of Medicine using frozen sections from mouse testes at two different ages (2-week-old and 8-week-old, n = 2). Mouse *Fance* coding sequences from exon 2 to exon 10 were amplified by RT-PCR using primers 5’-ggatgtgtcctctaccactgatg (sense) and 5’-cctggatggactgtcttcttt (antisense). The PCR product was ligated into a TOPO vector (pCRTMII-TOPO vector, Cat# K4600-01, Invitrogen). Linearized plasmid was used for in vitro transcription to incorporate digoxygenin-labeled UTP (DIG RNA Labeling Kit, Cat#1175025910, Roche). Sense and antisense probes were generated using SP6 or T7 polymerase, respectively.

### Hematoxylin and eosin (H&E) staining

Testes were placed into Bouin’s solution immediately after dissection, and kept in the solution for 6 hours at room temperature (RT). Tissues were rinsed in 70% ethanol before paraffin embedding. Testes were sectioned at a thickness of 5μm and the sections were stained with H&E.

### TdT-mediated dUTP nick-end labeling (TUNEL)

TUNEL analysis was performed on paraffin-embedded testes sections using the In Situ Cell Death Detection Kit, Fluorescein (Roche, Cat#11684795910) according to the manufacturer’s instructions. Sections were mounted and counterstained with Vectashield containing DAPI (Vector Laboratories, H-1200). A positive control slide was prepared by incubation with DNaseI(Sigma, D-4527) for 30 min at room temperature. The negative control slide received 2ul of distilled water in place of 2ul of TdT2 in the fluorescein-12-dUTP reaction mixture.

### Spread chromatin analyses

Germ cell chromatin spreads were prepared as previously described [22]. The primary antibody to SYCP1 (NB300-229, Novus Biologicals) was diluted 1:500 and the SYCP3 antibody (sc-74569, Santa Cruz) was diluted 1:50. Images were captured using a Zeiss CellObserver Z1 linked to an ORCA-Flash 4.0 CMOS camera (Hamamatsu) and analyzed with the Zeiss ZEN 2012 blue edition image software.

### Immunohistochemical staining

After deparaffinization and rehydration, the testicular sections were heated for 15 min in a microwave oven at 100°C with sodium citrate buffer (10mM; pH 6),cooled down in the same buffer at RT, then incubated for 20 min with 3% hydrogen peroxide. After washing with phosphate-buffered saline (PBS), the sections were incubated with primary antibodies in a humid chamber at 4°Covernight, then washed again three times in PBS. Primary antibodies were detected by incubation with 1:300Biotin-XX Goat Anti-rabbit IgG (H+L)(Life Technology, B-2770) or Biotin-XX Goat Anti-rat IgG (H+L)(Life Technology,A-10517) for 1 hr at RT, then horseradish peroxidase Streptavidin(SA-5704,Vector) was added. The peroxidase activity was made visible with Vector NovaRED^™^ (SK-4800) and counterstained with hematoxylin for 10 sec to 2 min.

Negative controls omitting primary antibodies were included in each experiment. Primary antibodies and dilutions used are presented in Table 1.

**Table 1:** Primary antibodies used in this study.

Antibody	Host	Source	Catalogue number	Dilution
**TRA98**	rat	Abcam	Ab82527	1:200
**FANCD2**	rabbit	Novus Biologicals	NB100-182	1:100
**Phospho-H2AX(Ser139)**	mouse	Biolegend	613401	1:100
**CHK1**	rabbit	BethylLaboratories	IHC-00004	1:100

### Statistics methods

The data are presented as mean ± standard deviation. The differences in relative transcript levels of *Fance* mRNA, and weights of body and testes were compared by independent sample t-tests using SPSS software (version 16.0). P value <0.05 was defined as statistically significant.

## Results

### Assays of *Fance* transcription in homozygous mutant (MT for short) male mice

**Fig 1:**
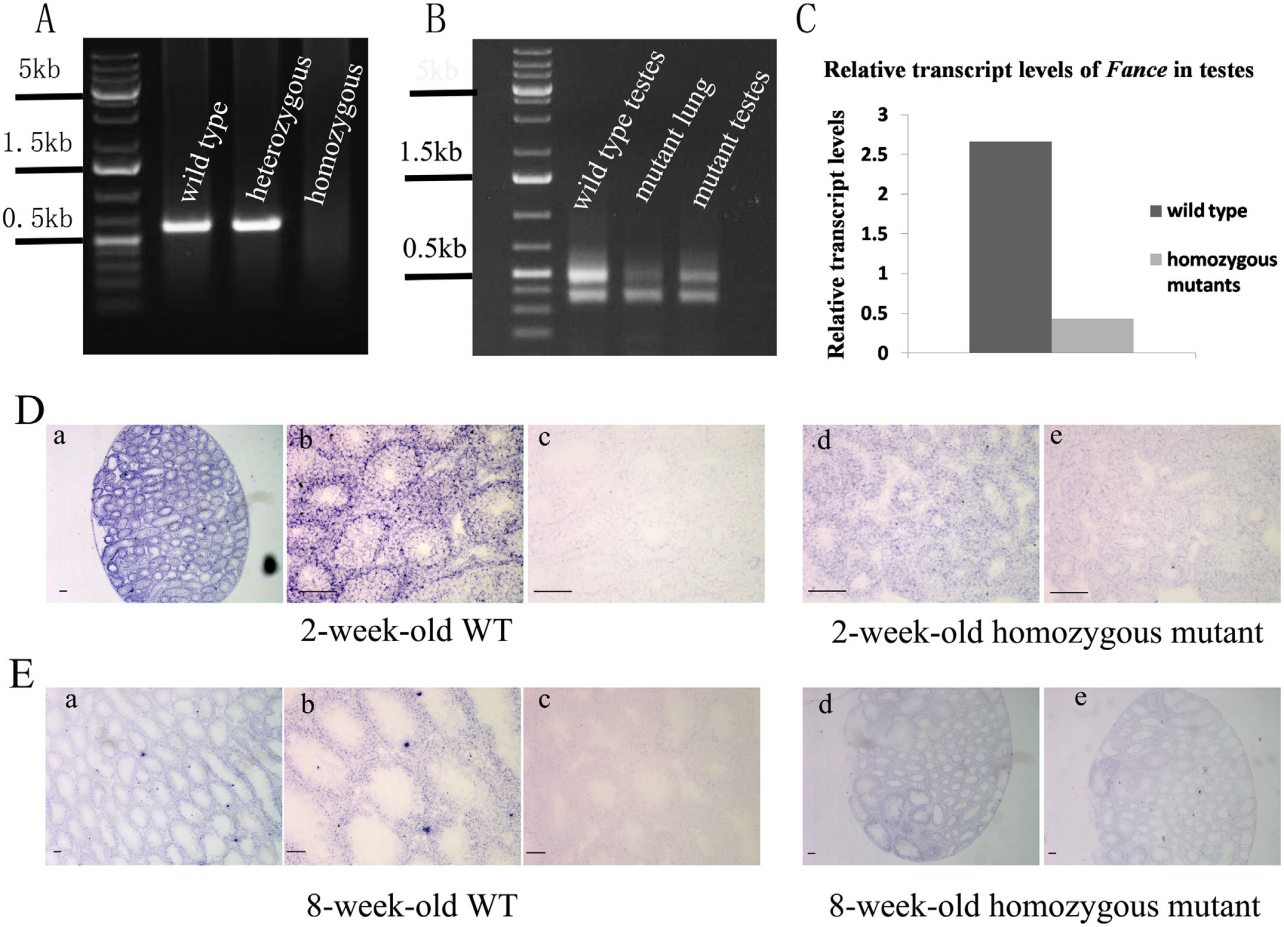
Decreased levels of Fance transcription in homozygous mutant males. (A) PCR genotyping of mouse tail DNA. PCR amplifications were performed using primers that flank the lentiviral integration site. Homozygous mutants showed no amplification band. (B) RT-PCR analysis of *Fance* expression. Using a sense primer from exon 6 and an antisense primer from the last exon (exon 10), RT-PCR amplifications produced 2 bands for RNAs isolated from wild-type or mutant tissues. Sequencing of the wild-type bands revealed that the two bands were produced from transcripts with (upper band) or without (lower band) exon 9. Sequencing of the bands amplified from the *Fance* MT revealed splicing defects for both bands and the loss of an open reading frame (data not shown). (C) Relative transcript levels assayed by qRT-PCR. (D) and (E) *In situ* hybridization assays using 2-week-old and 8-week-old mouse testes (Scale bars = 100μm). The antisense probe (panels a, b) detected *Fance* transcripts at both ages, with evidence for higher levels of transcript at 2 weeks of age. A sense probe (c) did not produce a positive hybridization signal. In the homozygous mutant testes, no signal above background was detected using either an antisense probe (d) or a sense probe (e).

We used both darker coat color (not shown) and genomic PCR assays to identify homozygous mutant mice. Using primers that flank the lentiviral integration site, no amplification band was detected in *Fance* homozygous mice by PCR (Fig 1A). We used 3 different protocols to assay for changes in *Fance* expression at the RNA level. Decreased *Fance* transcripts were detected by RT-PCR (Fig 1B). DNA sequencing showed that the top band contained exons 6–10 and the bottom band contained exons 6–8, and 10 in wild-type testes (data not shown). Both bands in the mutant testes had abnormal splice sites, resulting in loss of the open reading frame (data not shown). By qRT-PCR, relative transcript levels of *Fance* mRNA were significantly reduced in homozygous mouse testes compared with those in WT mice(P<0.01, n = 8 each group, Fig 1C). Using *in situ* hybridization, *Fance* expression was detected in seminiferous tubules and stromal cells of WT testes, but was not detected above background in *Fance* MT testes at 2-weeks of age or 8-weeks of age (n = 2, Fig 1D and 1E).

### Phenotypic abnormalities in *Fance* MT male mice

**Fig 2:**
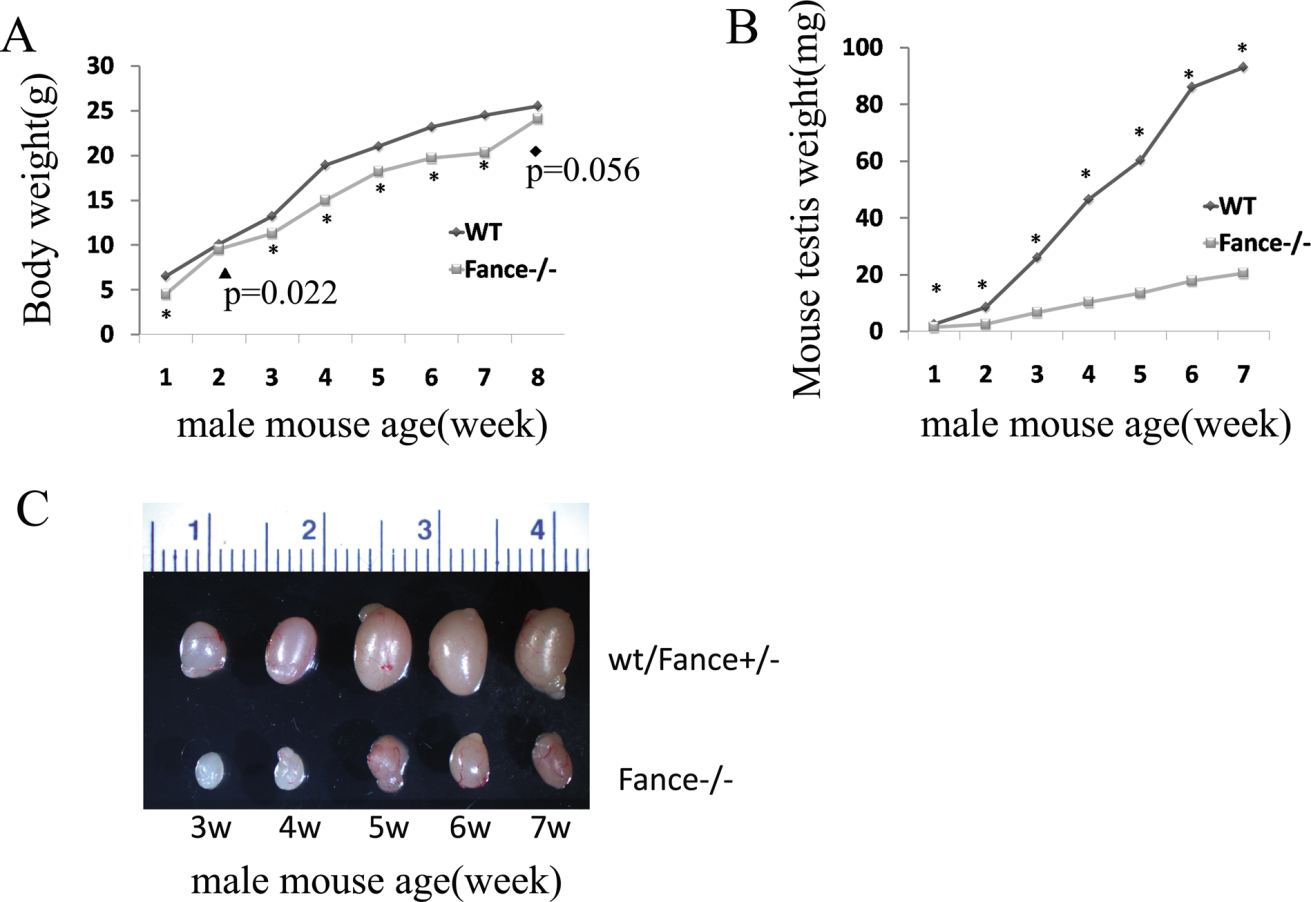
Phenotypic characteristics in *Fance* MT male mice. (A) Body weights of control (wt) and mutant (*Fance*^-/-^) male mice. Mutant mice weighed less than the control mice from 1 week to 7 weeks of age (*P<0.01). (B) Testes weights. Mutant testes weighed less than controls at all ages after 1 week of age (*P<0.001). (C) Testes photographs. *Fance*^-/-^ testes(bottom row)were visibly smaller than control mouse testes(top row).

Mutant male mice had body weights that were10-15% lower than their siblings beginning shortly after birth and lasting until two months of age (P<0.05, n = 8, Fig 2A). They also had a slightly higher rate of newborn lethality (data not shown). They did not show microophthalmia. We saw no evidence for enhanced tumor formation during one year of observation.

The mutant testes showed gonadal hypoplasia. The weights and sizes of the testes in *Fance* MT mice were significantly lower than in the control mice from 1 week to 8 weeks of age (P<0.001,n = 8, Fig 2B and 2C). The average testis to body weight ratio for the WT mice increased over time. In contrast, this ratio decreased over time for the *Fance* MT mice(P<0.001,n = 8, Table 2). *Fance* MT male mice showed impaired fertility. Only 5 out of 8 male mutants produced offspring. The average number of litters sired over 6 months was 4.1±0.8 for the control males versus1.4±1.4 for the MT males (P<0.001). The average litter size for control and *Fance* MT males was 8.4±2.0 and 7.6±3.0 respectively (P = 0.462).

**Table 2:** Testis to body weight ratios (%, n = 8 in each age group).

Mouse Age	WT	Fance MT
**1 week**	0.045	0.033
**2 weeks**	0.083	0.028
**3 weeks**	0.22	0.077
**4 weeks**	0.25	0.069
**5 weeks**	0.27	0.073
**6 weeks**	0.38	0.075
**7 weeks**	0.39	0.091

### A serious deficit of male germ cells in *Fance* MT mice

**Fig 3:**
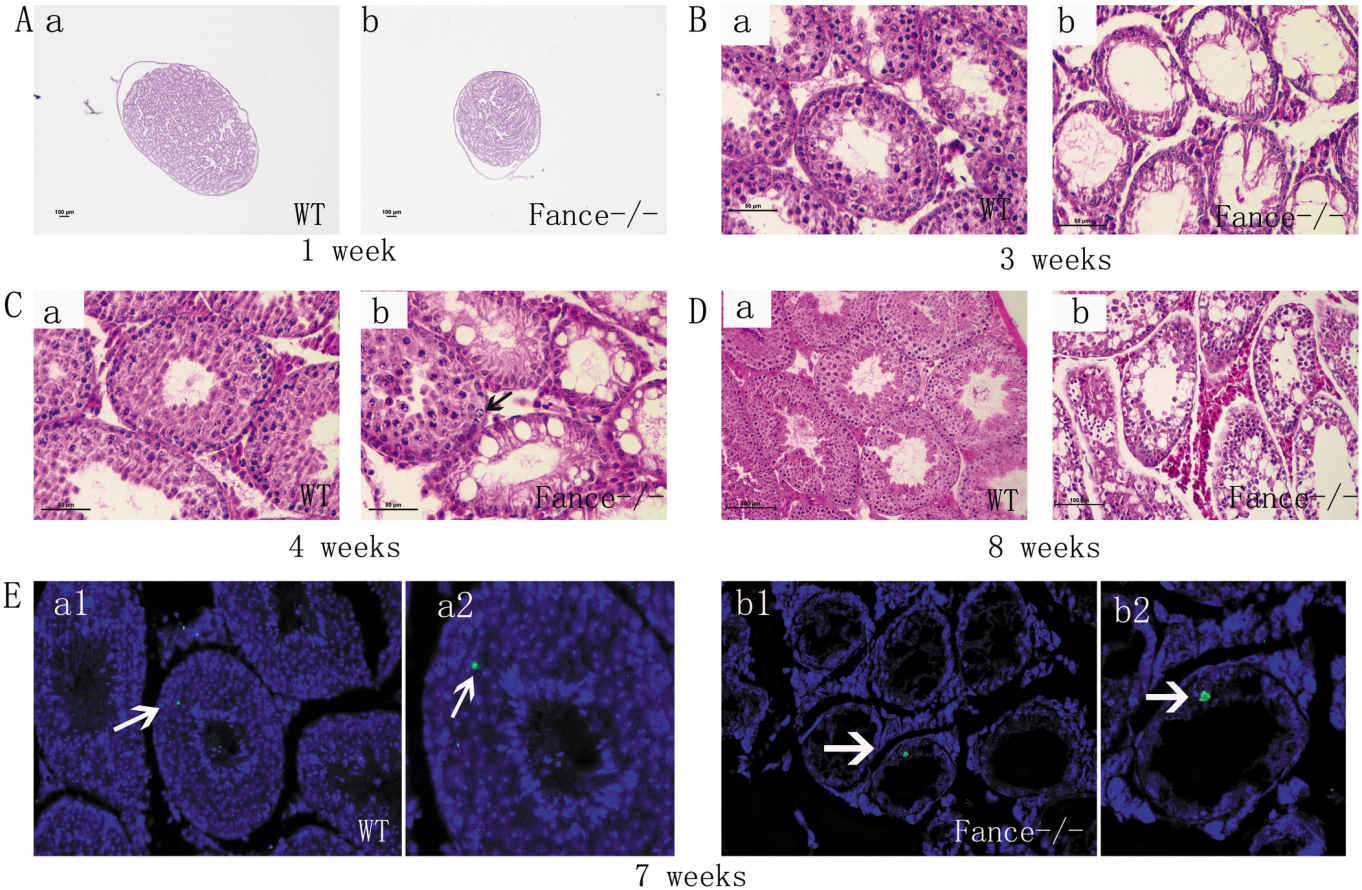
Testes histology and apoptosis. In each comparative panel, the “a” images are from control testes, the “b” images are from homozygous *Fance* mutant testes. (A)-(D)H&E stained paraffin sections from 1 week (A), 3 week (B), 4 week (C) and 8 week (D) old testes. Scale bars = 100μm in A and D, and 50μm in B and C. (E) TUNEL staining to detect apoptosis. Only one apoptotic cell (green) was detected in the WT and *Fance*^-/-^testes sections (white arrows). The sections were counterstained with DAPI (blue).

Histology sections revealed obvious changes in the testes of the mutant males (Fig 3). Between 3 weeks and 8 weeks of age, many of the tubules showed extensive vacuolization (n = 8, Fig 3B–3D). At 3 weeks of age, WT tubules contained multiple spermatocytes undergoing meiosis (Fig 3B). Very limited numbers of spermatocytes were seen in the mutant testes at this age (Fig 3B). By 4 weeks, round spermatids were present in the control males (Fig 3C). In contrast, most tubules in the mutants were still devoid of spermatocytes, but some tubules showed a cohort of primary spermatocytes undergoing meiosis (Fig 3C, arrow). By 8 weeks, the control testes showed mature sperm in some tubules, whereas mature sperm were rarely observed in the mutant testes at this age (Fig 3D). Apoptotic cells were rare in control and *Fance* MT testes at 7 weeks of age (n = 5, Fig 3E).

**Fig 4:**
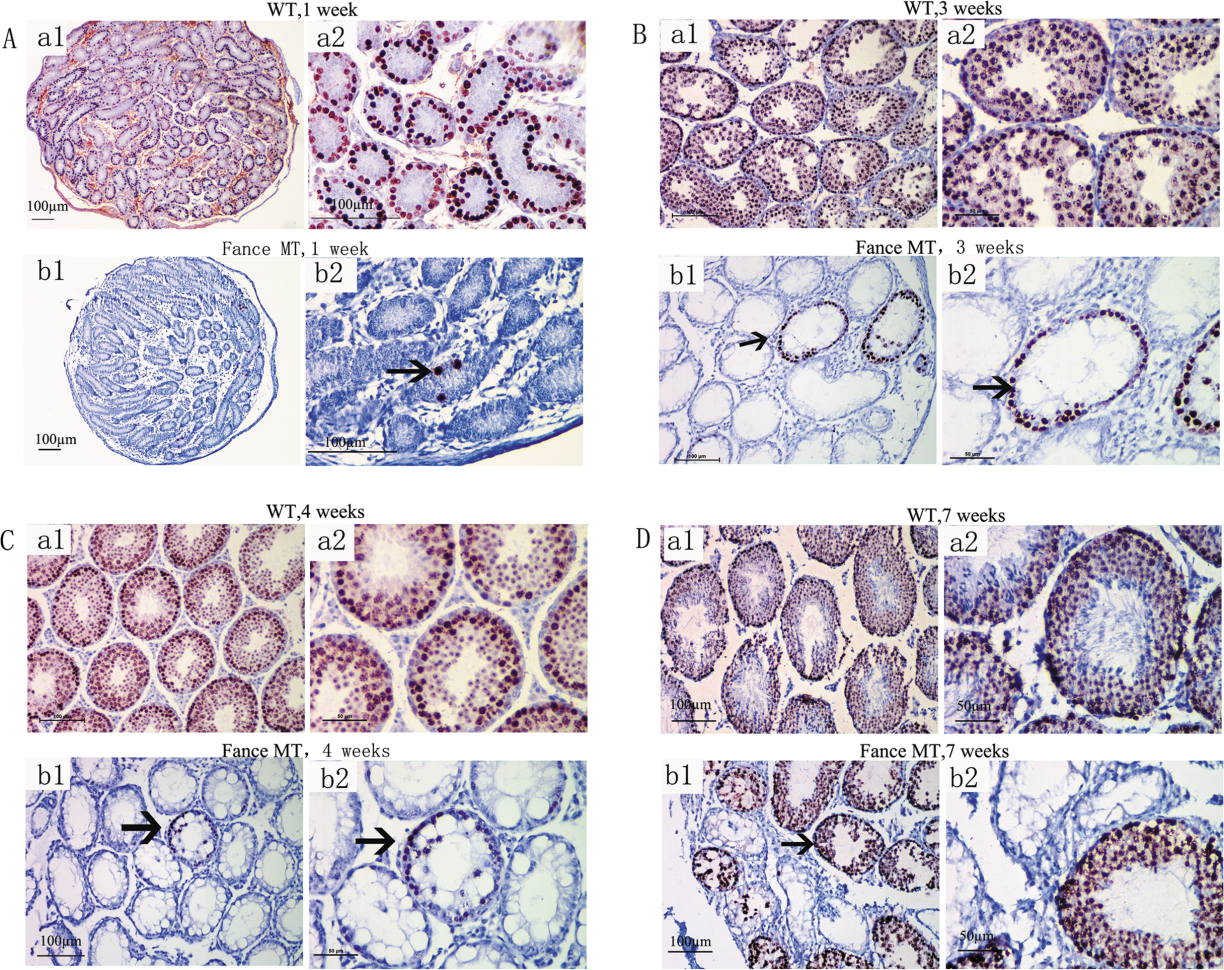
Severe germ cell deficiencies in *Fance* homozygous mutant testes. In each comparative panel, images a1 and a2 are from control (WT) testes, images b1 and b2 are from *Fance* MT testes. Sections from mouse testes at 1-week (A), 3-weeks (B), 4-weeks (C) and 7-weeks (D) of age were assayed by immunohistochemistry using the germ cell-specific antigen antibody TRA98. Nuclear TRA98 immunostaining (red precipitate, arrows) was seen in spermatogonia, spermatocytes and spermatids.

TRA98 is a germ cell specific antibody. The antigen for TRA98 is localized to the nuclei of testicular germ cells(Fig 4A–4D). TRA98 immunostaining was used to assess the presence and status of germ cells in the WT and mutant testes at different ages (n = 4 in each age group). At one week of age, TRA98-positive spermatogonia were detected around the periphery of each testes tubule in the WT testes (Fig 4Aa1 and 4Aa2). In contrast, there were only small numbers of positive cells in the mutant tubules, and positive cells were detected in only a subset of the tubules (Fig 4Ab2). By 3 and 4 weeks of age, maturing germ cells were present in abundance in all tubules in the control testes (Fig 4B and 4C). At these ages, some of the tubules in the mutant testes had germ cells that had expanded around the full periphery of the tubules. Later stages of germ cell maturation were not seen at this age in most of the tubules. By 7 weeks of age, spermatogenesis had begun in some, but clearly not all, of the mutant tubules (Fig 4Db1 and 4Db2).

### Reduced numbers of meiotic cells in testes of *Fance* mutant mice

**Fig 5:**
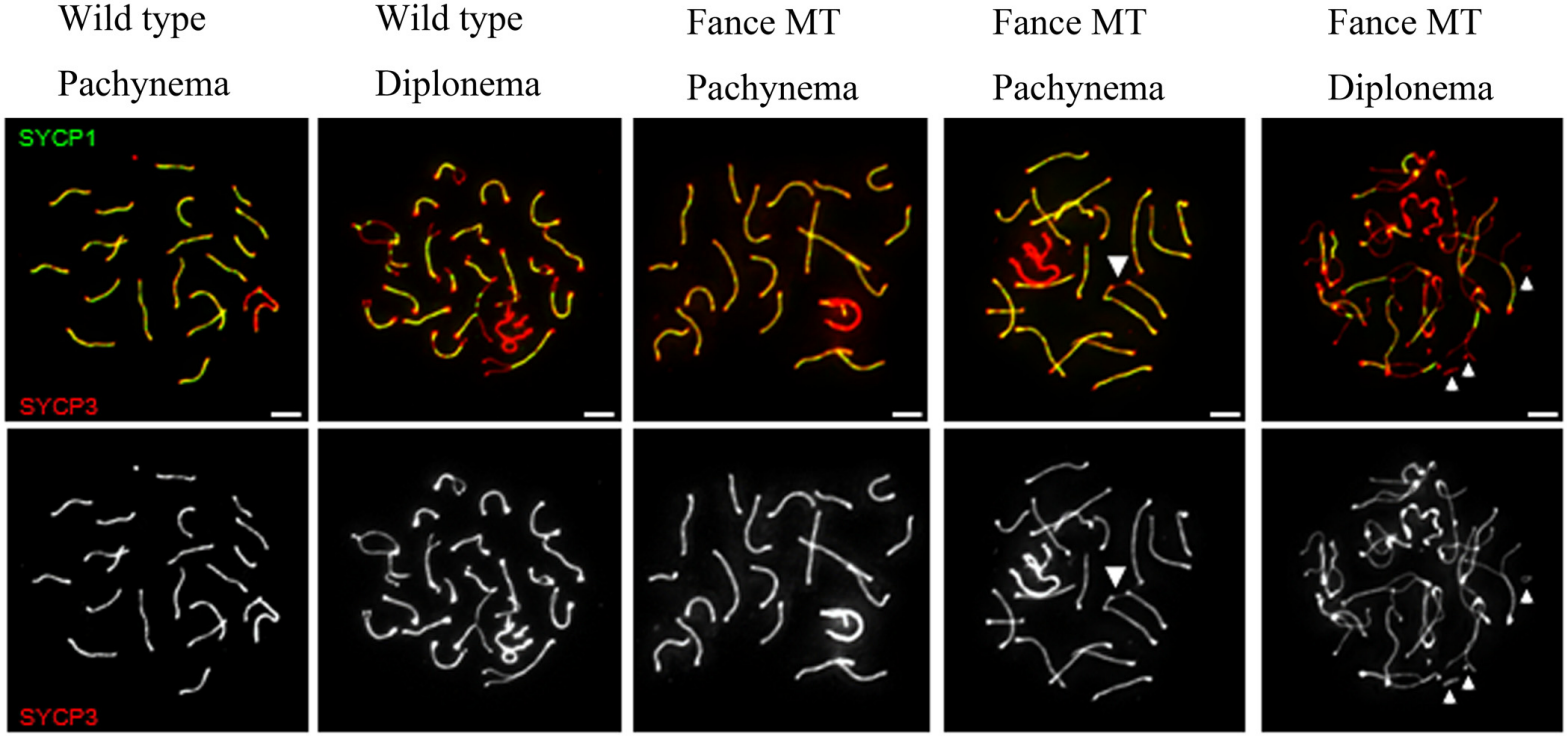
Meiotic spreads. Meiotic chromosome spreads from wild type and *Fance* mutant (MT) primary spermatocytes isolated from the testes at 20 dpp. Each chromatin spread was staged by analyzing SYCP3 (red) and SYCP1 (green), which are axial element and central region components of the synaptonemal complex respectively. Most of the *Fance* mutant spermatocytes displayed normal synapsis (middle panels); however a sub-set displayed synapsis abnormalities including an association between non-homologous chromosome ends (arrow head) and abnormal SYCP3 structures (arrows).

We processed 20 day post-partum (dpp) testes for chromatin spreads and immunostained using antibodies for the meiotic markers Synaptonemal Complex Protein 1(SYCP1) and SYCP3 (n = 2). The number of meiotic cells present in the mutant preparation was markedly less than the litter mate control. In the WT testes, 59% of the cells were positive for SYCP3 vs. 8.3% in the *Fance* MT. Previous work has reported meiotic defects in a subset of primary spermatocytes from other FA mouse mutants[12,23]. We found that most of the mutant spermatocytes exhibited normal synapsis (Fig 5, middle panels), but 6.5% (13 out of 200) of *Fance* mutant meiotic spermatocytes showed modest synapsis defects (Fig 5).

### Detection of phospho-H2AX and Chk1 in *Fance* MT testes

**Fig 6:**
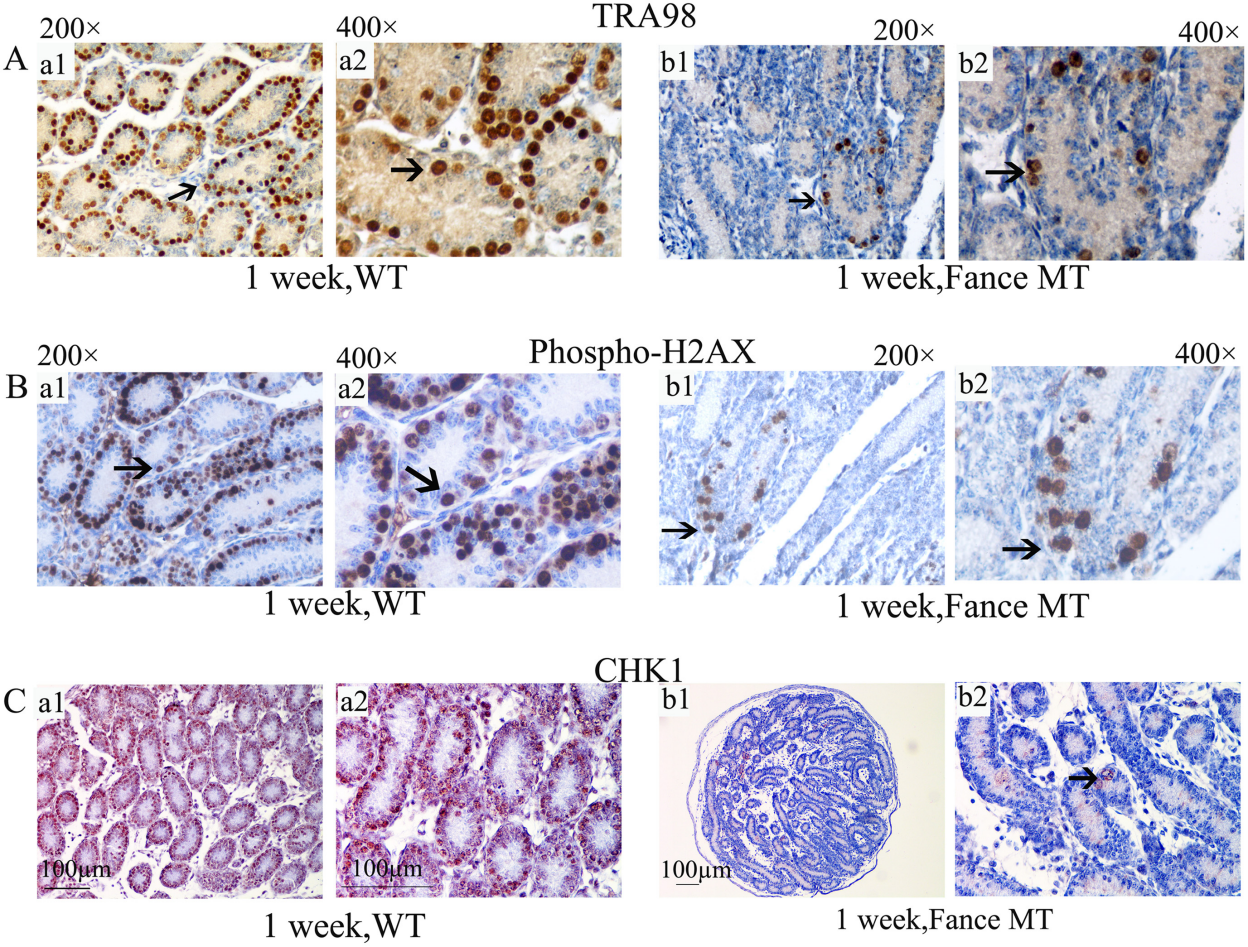
Immunohistochemical (IHC) detection of TRA98, phospho-H2AX and Chk1 in 1 week old testes. In each comparative panel, images a1 and a2 are from control (WT) testes, images b1 and b2 are from *Fance* MT testes. (A) IHC for TRA98. (B) IHC for phospho-H2AX. (C) IHC for Chk1. For each antibody, both control and mutant germ cells showed positive nuclear staining (indicated by arrows).

Since the FA proteins are involved in the repair of DNA damage, we predicted that the mutant germ cells might show significant alterations in expression or localization of proteins that are involved in the molecular detection of double-stranded DNA breaks, or in cell cycle regulation in response to unrepaired DNA damage. The histone family 2A variant (H2AX) becomes phosphorylated on Serine 139 in response to DNA damage, and also at the onset of meiosis [24, 25]. We found that the immunostaining patterns of phosphorylated H2AX were similar to those of TRA98 both in control and *Fance* mutants (n = 5, Fig 6A and 6B). Although there were far fewer germ cells in the mutant tubules, the germ cells that were present showed positive immunostaining (Fig 6Bb1). The kinase Chk1 is activated in response to DNA damage [26]. Chk1 expression was detected by immunostaining in the nuclei of most control germ cells at 1 week of age (n = 5, Fig 6Ca1 and 6Ca2). The few germ cells that were present in the Fance mutant testes at this age also showed positive nuclear immunostaining with the Chk1 antibody (Fig 6Cb1 and 6Cb2).

### Localized spermatogenesis in *Fance* MT testes

**Fig 7:**
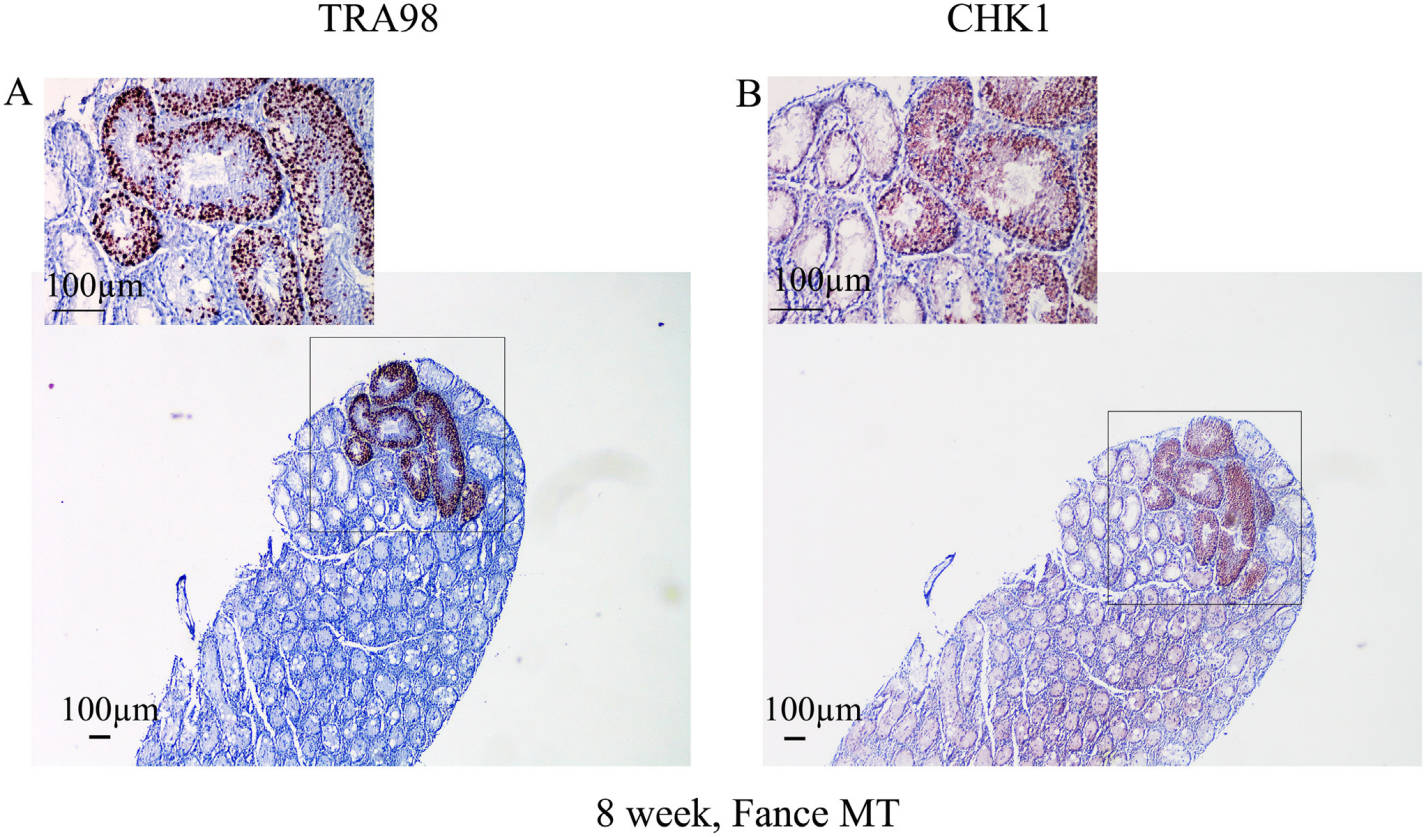
Localized germ cell maturation in 8 week old *Fance* MT testes. (A) IHC for TRA98. (B) IHC for Chk1.

A localized, mosaic, pattern of germ cell expansion within the *Fance* mutant testes was quite obvious by 8 weeks of age. Immunostaining of serial sections with the TRA98 and Chk1 antibodies showed that germ cells were present in only a localized set of tubules within the older testes, but within these tubules, maturation of the germ cells was proceeding normally, resulting in the production of mature sperm (Fig 7).

### Fancd2

**Fig 8:**
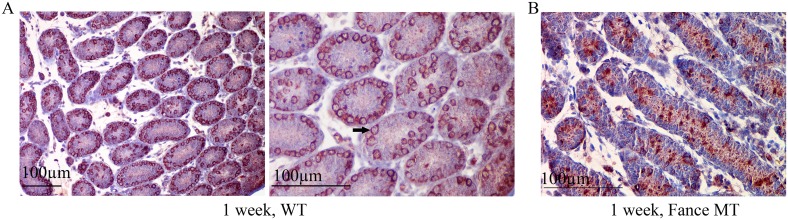
IHC forFancd2. (A, B) IHC for Fancd2 protein. Fancd2was localized around the nuclear membrane in the wild-type germ cells (arrows). No perinuclear staining was seen in the mutant testes.

Our attempts to detect the Fance protein by immunohistology were unsuccessful. However, immunostaining with an antibody to Fancd2 protein revealed a distinctive pattern of perinuclear accumulation in the germ cells of the 1 week old WT testes (n = 5,Fig 8A). We did not detect a similar pattern of staining in the 1 week old *Fance* mutant testes (n = 5,Fig 8B).

## Discussion

We describe herein a novel transgenic mouse line with an insertional mutation in *Fance*. We show that the lentiviral insertion in intron 8 results in defects in Fance transcription in the homozygous mutant testes. The phenotype of *Fance* MT male mice is reminiscent of other FA mutants [4–12]. In particular, the mice have a serious lack of male germ cells, leading to delayed fertility or infertility.

FA is an autosomal recessive disease in humans [27, 28]. FA patients have a heterogeneous clinical phenotype [29]. The characteristic cellular phenotype in FA patients is hypersensitivity to DNA cross-linking agents, which is the main basis for clinical diagnosis [30, 31]. Although the *Fance* mutant males show an initial stage of growth retardation (Fig 2), they eventually become the *Fance* mutant mice with a normal appearance and no obvious external malformations. At all ages, the *Fance* mutant mice have smaller than normal testes as well as a severe deficit of germ cells. In many of the mutants, there is a small population of testicular germ cells. These cells often manage to proliferate over time, eventually leading to the formation of functional sperm.

Our immunostaining assays suggest that the few germ cells that are present in the post-natal mutant testes have the molecular characteristics of normal germ cells. We did not detect elevated levels of apoptosis, although the frequency of cell death is difficult to quantify due to the initial death of germ cells. At the molecular level, we do not detect any obvious defects in the mutant germ cells, other than perhaps abnormal localization of the Fancd2 protein. We do not know whether Fancd2 becomes destabilized, or mislocalized, in the absence of the Fance protein.

Once the mutant germ cells have locally expanded to sufficient numbers to surround the circumference of a tubule, they proceed through all stages of normal spermatogenesis. We think that this phenomenon indicates that a minimum density of germ cells is needed in order to trigger entry into meiosis and progression to the later stages of spermatogenesis. Older fertile homozygous mutant males produce nearly normal sized litters when mated to WT or heterozygous mutant females.

From the perspective of maintaining the integrity of the genome, primordial germ cells may be constrained to replicate their DNA in a manner that introduces a minimal number of new mutations. Although NHEJ could be used to repair double-stranded DNA breaks that occur during replication, NHEJ is inherently mutagenenic and is therefore problematic. Consequently, NHEJ may be tightly regulated, or even inhibited, in primordial germ cells. Primordial germ cells may rely on the FA complex for effective repair of replication-associated DNA damage. When the FA complex is inactivated, cell cycle progression may become inhibited and germ cell proliferation may be stalled. The mutant primordial germ cells may somehow regain their proliferative capacity once they have migrated to the maturing gonad. In the gonad, germ cells may upregulate expression of certain proteins that are involved in NHEJ, which may be helpful in regaining their proliferative capacity. We provide this as a hypothetical model to explain why the small number of germ cells that are present in the mutant newborn testes can re-enter a proliferative phase and can progress through the later stages of spermatogenesis, even though their prenatal proliferation was significantly impeded. It is worth noting that we have seen no evidence for enhanced frequencies of mutagenesis in our *Fance* mutant line of mice. None of the offspring has shown a new mutant phenotype and none of them has shown any evidence of semi-sterility, which would be a consequence of chromosomal translocations.

In summary, our results indicate that *Fance*, just like other FA genes, is important for primordial germ cell expansion. Although very few germ cells are present in the 1 week old mutant testes, these mutant germ cells can proliferate within the testes tubules, and if they reach a critical density, can undergo all later stages of spermatogenesis, thereby producing mature, functional, sperm.
